# Cutting Forces in Peripheral Up-Milling of Particleboard

**DOI:** 10.3390/ma14092208

**Published:** 2021-04-25

**Authors:** Bartosz Pałubicki

**Affiliations:** Department of Woodworking and Fundamentals of Machine Design, Faculty of Wood Technology, Poznan University of Life Sciences, ul. Wojska Polskiego 38-42, 60-637 Poznań, Poland; bpalubic@up.poznan.pl

**Keywords:** particleboard machining, particleboard milling, cutting force, specific cutting force, chip thickness

## Abstract

An analysis of forces acting in the peripheral up-milling of particleboard is presented. First, a novel method of high-frequency piezoelectric force signal treatment is proposed and used to separate the original force signal from the vibrations of the previous cutting iteration. This allows for the analysis of single chip cutting force courses during industrial CNC (Computer Numerical Control) milling. The acting forces are compared with the theoretical, instantaneous, uncut chip thickness. The results show that, for a range of 40–60 m/s, the higher the cutting speed used, the higher the resultant and principal cutting forces. The method of cutting thrust force used was similar to that observed in solid wood milling, i.e., first using a pushing action, followed by a pulling action. The obtained average specific principal cutting forces for particleboard peripheral up-milling are equal to 32.0 N/mm^2^ for slow and 37.6 N/mm^2^ for fast milling. The specific cutting thrust force decreases with the increase in instantaneous uncut chip thickness.

## 1. Introduction

Peripheral milling is a common method for processing wood and wood-based materials utilized for planers, thicknessers, moulders, routers, etc. The process relies on cutting a single comma-shaped chip repetitively to create a surface with cut marks in the form of waves. The process itself is complex. It depends on the tool–workpiece interaction, which is related to the tool and workpiece properties, geometries and kinematic parameters such as cutting, feed speed, depth and width of cut, etc. The process produces a quality machined surface, as well as sawdust, noise and vibration. Energy is required to perform the process. The cutting energy distribution was carefully analyzed in [1], the most significant part being pure cutting, which can be analyzed in terms of cutting forces.

The process of cutting wood and the subsequent forces have been widely studied for decades. Different aspects have been of interest: wood species [2,3], moisture content [4], grain angle [5,6], age [7]. Multiple machining types, processing parameters, tool geometries and wear have been utilized for experimental and modeling work. Cutting forces have been measured directly by means of strain gage dynamometers [8] or with the use of more advanced piezoelectric sensors, since they are more suitable for dynamic processes. Often, cutting forces are found indirectly by measuring electrical power in the machining process [4,9].

Several cutting setups have been tested to determine cutting forces. A simple linear or quasi–linear (arc with a large radius) one-directional cutting movement allows for an easy and reliable investigation of the cutting process. Eyma et al. [10], as well as routing, took advantage of a pendulum to measure cutting forces. Since only one cut is performed at a time using this set-up, there is no impact from the vibration of the previous cut. The disadvantage of using a pendulum is a lower cutting speed, compared to industrial processes. This problem has been solved by Dvoracek et al. [11], who built a rotary 4 m diameter arm device, which is able to cut at a 100 m/s cutting speed and is also equipped with a force and strain measurement system. These methods are useful for obtaining basic knowledge of the material and process properties. Similarly, investigations on industrial machining processes such as rotary cutting peeling, band, sash gang, circular sawing, drilling, milling, etc., have been carried out [6,12,13,14].

The cutting processes of particleboards (PB), despite their great popularity, have not been investigated as much as solid wood machining. PB is a wood-based material with a characteristic structure of multiple wood chips of different sizes, glued together to form a panel, which is usually finished on both sides with a melamine coating. Compared to solid wood, PB has better shape and dimension stability, and even in-plane mechanical properties (transversal isotropy) do not cause macro-defects (such as knots in solid wood). These properties make PB very useful for cabinet production. When PB is subjected to the impact of a cutting edge, its structure disintegrates differently than in the case of a solid wood cutting process. It was noticed [15] that in PB cutting, pulverization of the chip occurs, especially for tools with small rake angles, which increases the risk of air contamination with fine, inhalable dust [16,17]. PB, together with medium-density fiberboard (MDF), is very often used in machining experiments of tool wear behavior, due to their highly abrasive properties [18,19,20,21]. Wong [15] modeled PB as an aggregate of wood chips and resin to investigate the cutting process. Three possible actions of cutting, namely particle crushing, splitting and tearing-out from the structure, were observed. It was also indicated that PBs differ significantly between manufacturers, due to variations in particle size, resin type and content, all of which can change the action and forces of cutting. Wong modeled cutting forces by assessing pressures in different zones of blade–workpiece interaction. Wong investigated “slow cutting” of PB, with cutting speeds that were much lower than industrial cutting speeds. Boucher et al. [22] modeled the different helix angles of the cutting edge during the milling of MDF and PB, but did not provide information on the cutting speeds used, stating that the spindle speed was lowered to 1000 rpm, because of occurring vibrations. When closer to industrial PB cutting speeds were used (around 24 m/s) [23], the vibrations of the workpiece and holder forced researchers to apply low-pass filtering, which distorted and decreased the force signal. Cutting forces were also measured for a turning operation of PB; however, this operation is not used industrially [24]. Again, all cutting speeds utilized by the authors were below 30 m/s. 

A common problem for all investigations into industrial PB cutting, except for those which are model-based, is the presence of strong vibrations. High cutting speeds generate vibrations not only at the machining center [25] but also at the fixed workpiece, significantly disturbing the force measurement. For rough measurement of the feed force that is felt by an operator during PB sawing [26], these vibrations do not play an important role, since several teeth are working at the same time, and the impact frequency is too high to be felt by the operator. However, in high-frequency measurements of cutting forces, these vibrations can introduce a significant error. The goal of this research is therefore to analyze the cutting forces during PB milling at full industrial speed, by applying a novel force signal treatment which ensures high reliability.

## 2. Materials and Methods

### 2.1. Materials

Wood based material in the form of one-layer PB was subjected to the CNC peripheral up-milling process. Pine wood core layer flakes with urea–formaldehyde resin with a 9% glue rate were used to produce four panels. A pressing temperature of 200 °C and a pressing time of 110 s were used.

Four specimens of nominal dimensions 100 mm × 60 mm and thickness *b* = 17 mm were cut out, each from a different panel. They were kept in a conditioning room (temperature 21 °C and air humidity 65%) for a week to obtain their constant mass. Density of the specimens was equal to 542.5 ± 6.8 kg/m^3^. 

### 2.2. Machine Tool

The Venture 115 M (Homag, Schopfloch, Germany) with a 10 kW spindle—a gate construction CNC machining center—was used as a machining tool. The workpieces were mounted with two M8 screws to an aluminum holder. The holder was fixed to an aluminum baseboard through a piezoelectric force sensor; the baseboard was mounted on the machine’s vacuum cups on the console table.

### 2.3. Cutting Tool

**Figure 1:**
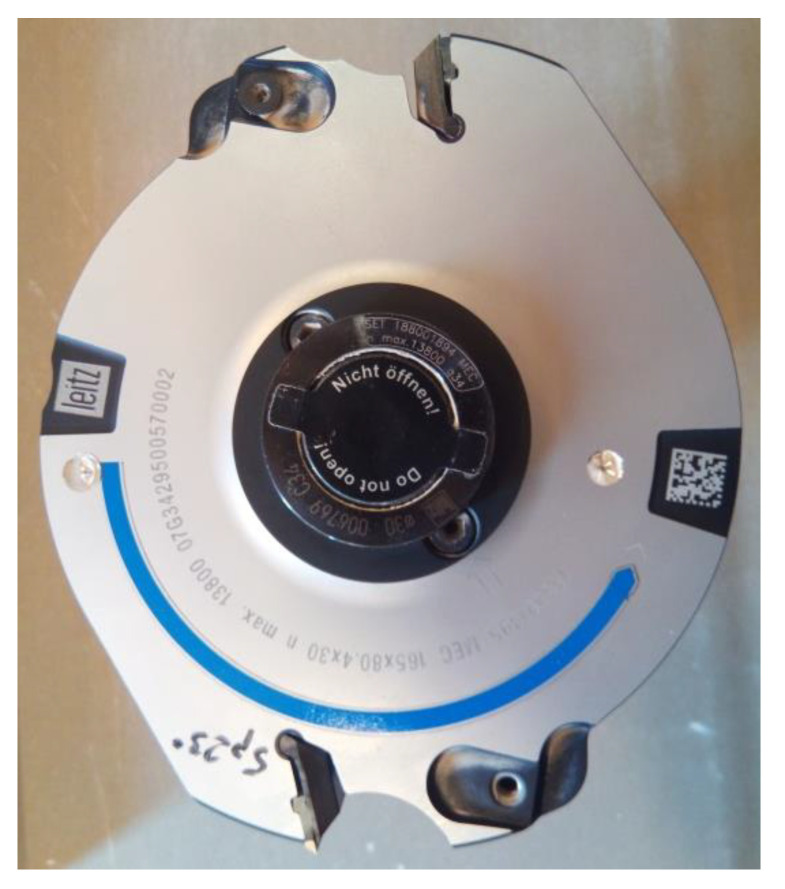
Milling head used in experiment.

A special HSK63 mount milling head (Leitz, Riedau, Austria) of 165 mm diameter (cutting radius *R* = 82.5 mm) was used in the experiments (Figure 1). Originally, it was a double blade tool, but for the current use, one of the blades was shortened, so that it was excluded from the cutting process. Therefore, only one (*Z* = 1) new, sharp, tungsten carbide cutting insert was used in the experiment. The geometry parameters of the cutting knife were as follows: clearance angle 20°, sharpness angle 57° and rake angle 13°. The tool wear was assumed to be negligible during the experiment, due to small total kerf length. The cutting edge was situated parallel to the axis of rotation. The tool was dynamically balanced.

### 2.4. Machining Process

**Figure 2:**
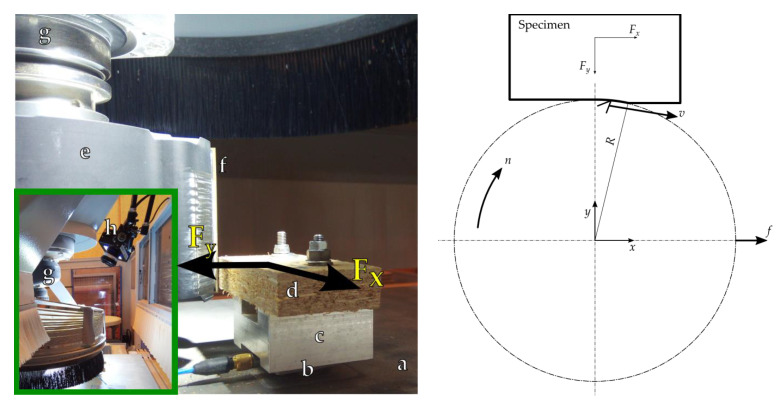
Experimental setup. General view (**right**-hand side): a—base board, b—force sensor, c—workpiece holder, d—workpiece, e—milling head. f—cutting edge, g—electro spindle, h—high-speed camera. Scheme (**left**-hand side) showing the directions of movements, coordination system of the position and of the force measurement during the up-milling process.

In the experiment, six up-milling operations (Figure 2) were performed, for two cases: slow and fast cutting. Cutting speeds *v*_1_ = 40 m/s and *v*_2_ = 60 m/s were achieved with two tool rotational speeds: *n*_1_ = 4630 min^−1^ and *n*_2_ = 6945 min^−1^. The constant feed per tooth *f_z_* = 1.5 mm, equal to the feed per revolution (since *Z* = 1), was obtained using two feed rates: *f*_1_ = 6.94 m/min and *f*_2_ = 10.41 m/min. The uncut chip geometry was identical for both cases. The depth of cut for both cases was *H* = 2 mm. 

**Figure 3:**
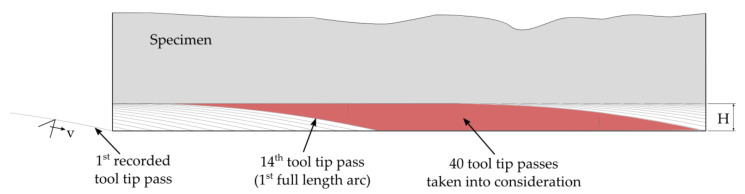
Subsequent tool tip passes in the workpiece. The first 13 passes at the beginning of machining were omitted from analysis, and the next 40 single full arc length cuttings were taken into consideration for force analysis (red color).

Depending on the initial position of the blade and the workpiece, only the 12th or 13th blade pass achieved full depth of cut (2 mm) and therefore resulted in a full, regular cutting force response (Figure 3). The same number of cuts at the end of one pass does not have a full arc length (18.95 mm) contact. In the present case, only 41 or 42 cuts in the middle region of the specimen were regular cuts, with full contact angle 12°38′. Due to the imperfections in the geometry of the measurement system, it was decided to reject the first 14 passes which produced any force response, and take into account the 40 knife impacts of each milling process. This yielded 240 single cuts to analyze for both slow (*v*_1_) and fast (*v*_2_) cases. 

### 2.5. The Force Measurement System 

The force measurement system consisted of PCB Piezotronics 260A02 (PCB Piezotronics, New York, NY, USA) piezoelectric sensor, PCB Piezotronics 482C16 signal amplifier and 16-bit analog-digital converter NI 9215 with CompactDAQ acquisition platform (National Instruments, Austin, TX, USA). The equipment was used to acquire two perpendicular force values *F_x_* and *F_y_* in the perpendicular directions *x* and *y* (Figure 2). The force signal acquisition with sampling frequency $\;f_{s}=$ 100 kHz, as well its processing and analysis, were performed with LabView software (National Instrumens, Austin, TX, USA). 

### 2.6. Force Signal Processing

Since the cutting process is highly dynamic, its measurement often poses a problem for researchers, because it requires not only a sensitive and high-speed force measurement system but also a trustworthy signal processing method. The signal recorded contains not only the direct instantaneous values of the forces exerted by the knife on the specimen, but also the dynamic response of the specimen–holder set mounted on the piezoelectric sensor. During experimentation, when the cutting edge hits the PB and the chip is cut, the set will start to vibrate at its natural frequency, which will be picked up by the sensor. When the next tool–specimen contact occurs, the force signal is combined with the remaining vibration signal, which makes it difficult to evaluate. Researchers have dealt with this problem in several ways. Palmqvist [27], in order to decrease the cutting speed and reduce the vibration disturbances on the force signal, used polypropylene specimens instead of wood. Maradpour et al. [28] neglected the problem and treated the raw data as unaffected by vibrations, by eliminating the first section of the signal. Goli et al. [5] applied Butterworth low-pass filtering to the raw signal and obtained force data that, as stated, were not exact, but easy to analyze. Additionally, Iskra and Hernandez [29] used an analog low-pass filter and no other post-processing of the force signal. Sommer et al. [30] noticed that low-pass filtering decreased the real force peak height, whilst also widening it. This meant that it was still suitable for calculating the mean cutting force applied to mean chip thickness in contact zones. A transfer function, based on the falling ball impulse, was successfully used by Krenke et al. [31,32] to convert the raw force signal into a clear-cutting force signal.

**Figure 4:**
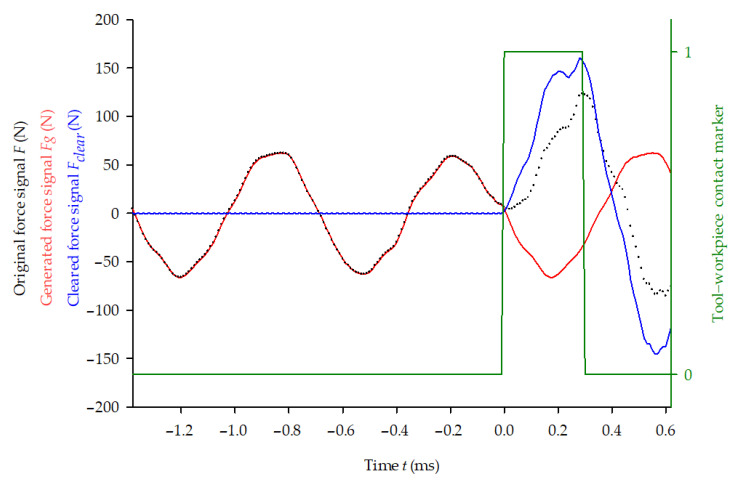
An example of the clearing procedure of the force signal: original force signal—black dots, generated force signal—red line, tool–workpiece contact marker—green line and cleared force signal—blue line.

In this study, it was decided to use a different approach. The original force signal was divided into smaller parts containing the cutting force peaks and preceding vibrations from previous tool impacts. The length of each signal portion was 25% of the tool impact period: 3.24 ms (*m* = 324 samples) for slow cutting (*v*_1_) and 2.16 ms (with *m* = 216 samples) for fast cutting (*v*_2_). Each portion of the force signal, after removing the part indicated by the tool–workpiece contact marker’s high value (Figure 4), was then decomposed with the use of the fast Fourier transformation algorithm into a discrete Fourier transform $\hat{F}$ in which any *k*-element is defined as a complex number:

(1) $${\hat{F}}_{k}=\overset{n-1}{\underset{j=0}{\sum }}F_{j}e^{-\frac{i\;2\pi \;jk}{n}}$$

where *F* is the original input force signal; *n* is the number of elements of *F.*

Next, the *Fg* signal was generated, imitating the original vibration signal *F*, but extended for all *m* elements (the entire portion of signal which also contained the cutting force peak). Each *j*-element of the newly generated signal was equal:

(2) $$Fg_{j}=\frac{2}{n}\overset{\frac{n-1}{2}}{\underset{k=0}{\sum }}\left|{\hat{F}}_{k}\right|\sin\left(\frac{k}{n}\;f_{s}\;j+\varphi_{k}\right)$$

where $\varphi_{k}=\arctan\left(Im\left({\hat{F}}_{k}\right):Re\left({\hat{F}}_{k}\right)\right)$.

Finally, the force signal cleared from vibration was calculated as $F_{clear}=F-F_{g}$, with its non-zero portion corresponding to the tool–workpiece contact (blue marker in Figure 4), which was extracted for further analysis. As visible in Figure 4, after the contact is finished, the cleared signal does not return to zero—the free vibration equilibrium has been disturbed by cutting, and the clearing procedure applied is not valid anymore. A new one must be performed before the next cutting force extraction. 

**Figure 5:**
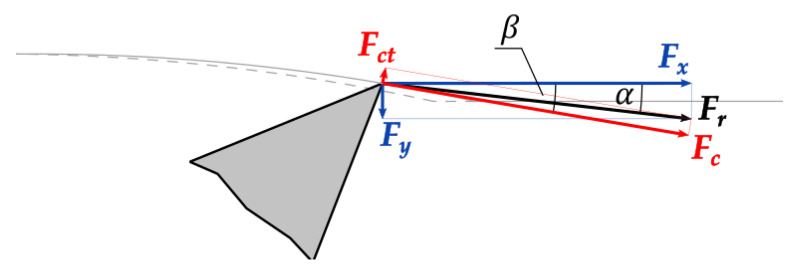
Forces considered in the cutting process. In the presented case, all forces and angles are positive.

The procedure described above has been applied to both *F_x_* and *F_y_* raw force signals. Then, the resultant force value *F_r_* shown in Figure 5 and the angle of its vector α have been derived as:

(3) $$F_{r}=\sqrt{F_{x}{}^{2}+F_{y}{}^{2}},\;\alpha =\arctan\left(\frac{F_{y}}{F_{x}}\right)$$

Finally, *F_r_* was decomposed into a principal cutting force $F_{c}$ working on the direction tangent to the cycloid curve (inclined by β) and a cutting thrust force $F_{ct}$ perpendicular to it:

(4) $$F_{c}=F_{r}\cos\left(\beta -\alpha \right),\;F_{ct}=F_{r}\sin\left(\beta -\alpha \right)$$

The $F_{c}$ is positive (+) when it is pointed in the direction of cutting speed vector *v*. The $F_{ct}$ is positive (+) when the workpiece is repelled by the tool, as defined in previous research [2]. As positive *F_x_* and *F_y_* directions are shown in Figure 2, the β angle is negative only at the very beginning of the tool–workpiece contact for the current setup and α may be positive or negative depending on *F_y_*.

### 2.7. Vision Control of the Process

During the tests, a high-speed camera, MotionXtra NX7 Speed 2 (Integrated Design Tools Inc., Pasadena, CA, USA), was used to observe the tool interaction with the workpiece. The camera and lighting were located above the workpiece and the tool. They captured frames at 15 kHz frequency, with an exposure time of 7 μs. 

## 3. Results and Discussion

### 3.1. Resulting Cutting Force

**Figure 6:**
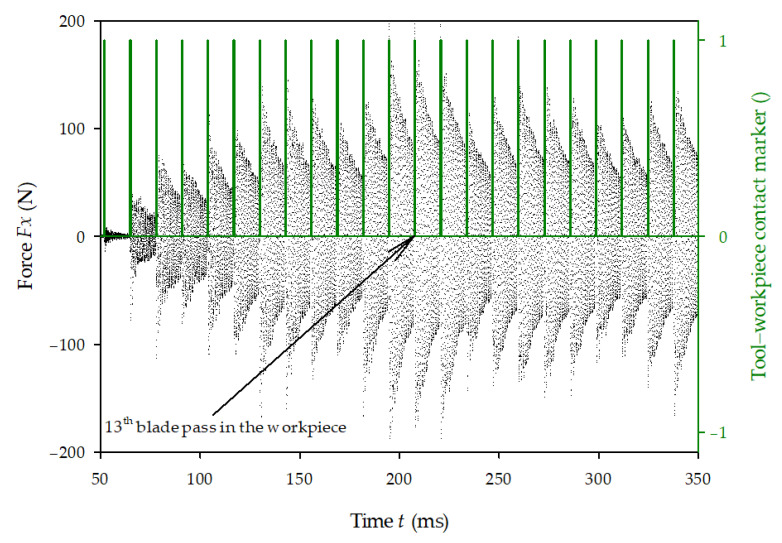
Raw force *F_x_* signal (black dots) and tool impact indicators (green lines).

An example of a raw force *F_x_* signal is shown in Figure 6. At the beginning, a small signal represents the first tool–workpiece contact, before the peak force amplitudes increase, along with the instantaneous depth of cut, whilst the cutting cycloid becomes more horizontal. After 13 impacts, the cutting process reaches full arc length and the force peaks stabilize. Some modulation of the signal was observed, due to the signal caused by vibration, which resulted in a phase shift of the signal, in relation to the force peaks.

**Figure 7:**
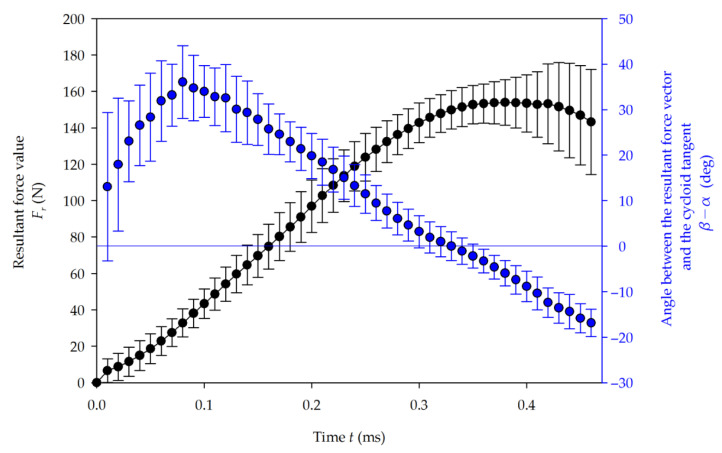
Average resultant force value *Fr* (black) and its angle α (blue) in the function of time for slow cutting.

**Figure 8:**
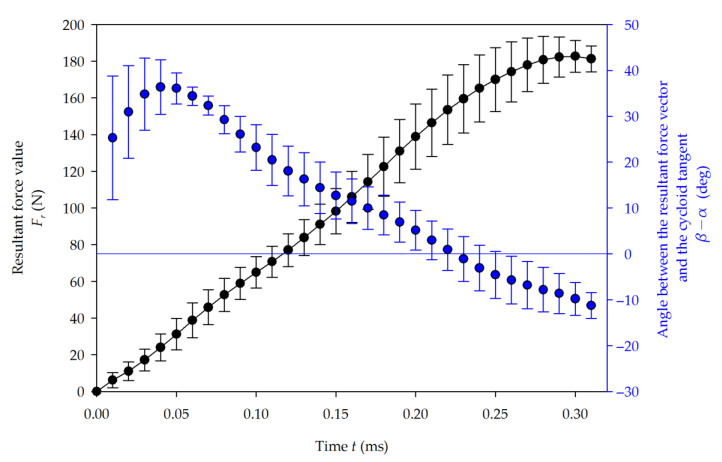
Average resultant force value *Fr* (black) and its angle α (blue) in the function of time for fast cutting.

The resultant force *F_r_* and angle α were calculated using Equation (3), and averaged. The slow cutting and fast cutting results are shown in Figure 7 and Figure 8, respectively. The maximal value of the resultant force was 153.9 N for slow cutting. In fast cutting, it was 182.7 N, which is 19% higher, where the cutting speed had been increased by 50%. In the slow cutting test, a more gradual flattening of the force curve is observed during the final stage of the tool–workpiece contact, as well as an increase in the standard deviation. The nature of this phenomenon is explained further by decomposing the resultant force into principal and thrust cutting forces.

At the beginning of the cutting process, the average inclination between the *F_r_* vector and the cycloid tangent direction (β−α) increases due to the pushing action of the clearance face onto the workpiece. Soon after, the tool tip plunges into the material, and the growing chip thickness causes an increased pulling action on the workpiece. This slows down the increase in the change of angle and, after 17.4% of the single contact time in the slow milling, the (β−α) angle reaches its maximum at 36°. For the fast process, a maximum angle of 34° is demonstrated after 15.6% of the total contact time.

### 3.2. Instantaneous Uncut Chip Thickness

To examine the cutting process, it is best to calculate the principal and thrust cutting forces, as previously defined. Additionally, in order to compare the forces that act during both slow and fast milling, it is best to consider them relative to the instantaneous uncut chip thickness (IUCT), instead of time (which is different for the fast and slow processes). Therefore, the IUCT should be derived as a function of the horizontal position *x* of the tool tip *h*(*x*). Many models of uncut chip thickness have been proposed so far: starting from simple circle-based paths, omitting the feed movement, through to more realistic models [33] and finishing with complex models that also take into consideration the tool run-out [34]. This, however, does not apply to the current case, since the tool is a large milling head. The theoretical model of IUCT *h*(*x*) for up-milling that is used in the present work was determined from the two following cycloid paths in the cutting zone, within the depth of cut. The origin of the coordinate system used is situated at the rotational axis of the tool at the moment in time when the *y* position, in the current cycloid, reaches its maximum *R* value. 

Each pass of the tool tip is a cycloid curve and may be presented as two parametric tool tip position functions of time (*t*), given in seconds:

(5) $$\{\begin{array}{c} x_{c}\left(t\right)=R\;sin\left(\frac{2\pi n}{60}t\right)+\frac{1000\;f}{60}t\;\\ y_{c}\left(t\right)=R\;cos\left(\frac{2\pi n}{60}t\right)\; \end{array}$$

where all parameters are in units as defined above. 

Since, in peripheral milling, only a part of the curve is involved in chip cutting, the single cutting edge pass may be represented by a $y_{c}\left(x\right)$ function instead, defined for the tool–specimen contact zone $x\in \langle -\frac{f_{z}}{2},\;x_{out}\rangle$, where $x_{out}$ is located at the point of the tool tip exit from the workpiece.

(6) $$y_{c}\left(x\right)=\sqrt{R^{2}-{\left(x-\frac{f_{z}}{2\pi }\arcsin\left(\frac{x}{R}\right)\right)}^{2}}$$

Its first derivative equals:

(7) $${y_{c}}^{\prime }\left(x\right)=\frac{\left(1-\frac{f_{z}}{2\pi r\sqrt{1-{\left(\frac{x}{r}\right)}^{2}}}\right)\left(\frac{f_{z}}{2\pi }\arcsin\left(\frac{x}{r}\right)-x\right)}{\sqrt{r^{2}-{\left(x-\frac{f_{z}}{2\pi }\arcsin\left(\frac{x}{r}\right)\right)}^{2}}\;}$$

and the precedent tooth pass function is:

(8) $$y_{c-1}\left(x\right)=\sqrt{R^{2}-{\left(x+f_{z}-\frac{f_{z}}{2\pi }\arcsin\left(\frac{x+f_{z}}{R}\right)\right)}^{2}}$$

For this case, the line covering the chip thickness direction is perpendicular to the tangent of the *y_c_*(*x*) curve at *x_i_* and contains (*x_i_*; *y_c_*(*x_i_*)) point. This intersects the previous iteration cycloid at point (*x_j_*; *y_c−_*_1_(*x_j_*)). The following equations must be solved:

(9) $$\{\begin{array}{ll} \frac{x_{j}}{y_{c}^{'}\left(x_{i}\right)}-y_{c-1}\left(x_{j}\right)-y_{c}\left(x_{i}\right)-\frac{x_{i}}{y_{c}^{'}\left(x_{i}\right)}=0, & \;|x_{i}\in \langle -\frac{f_{z}}{2},0\;)\cup (0,\;x_{j\;out}\rangle \\ x_{j}=0,\; & |x_{i}=0\\ \frac{x_{j}}{y_{c}^{'}\left(x_{i}\right)}-\left(R-h\right)-y_{c}\left(x_{i}\right)-\frac{x_{i}}{y_{c}^{'}\left(x_{i}\right)}=0,\; & |x_{i}\in (x_{j\;out},\;x_{i\;out}\rangle \end{array}$$

The IUCT was calculated iteratively, as in the work [35]. At a specific *x_i_* position, it is equal to:

(10) $$h\left(x_{i}\right)=\sqrt{{\left(x_{i}-x_{j}\right)}^{2}+{\left(y_{c}\left(x_{i}\right)-y_{c-1}\left(x_{j}\right)\right)}^{2}}$$

**Figure 9:**
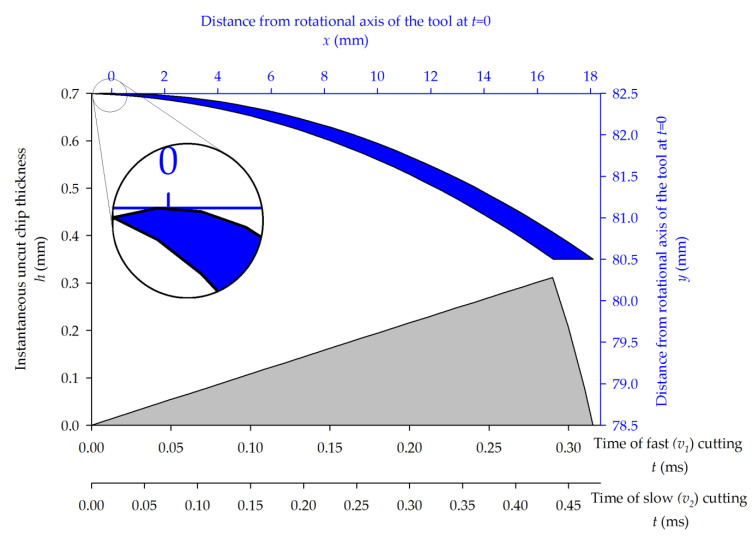
Shape of the uncut chip (blue) given in two dimensions and its calculated instantaneous thickness (gray).

Figure 9 shows the shape of the uncut chip and its calculated instantaneous thickness. As visible (also from Equation (9)), near the end of the cutting processes, the chip thickness decreases because the blade is exiting from the board. To analyze cutting forces in relation to the increasing IUCT, it was decided to consider data sets from up to 0.29 ms for fast and 0.44 ms for slow milling. The maximal chip thickness was then shown to approximately equal 0.31 mm.

### 3.3. Principal and Thrust Cutting Forces

From the literature, the influence of the cutting speed on the cutting force is not clear. For pine wood, it was found [36] that for higher cutting speeds, from the range of 15–40 m/s, both principal and thrust cutting forces slightly increase. Previous research demonstrates an opposite relationship [37]. In addition, [38,39] point out a clear correlation. 

**Figure 10:**
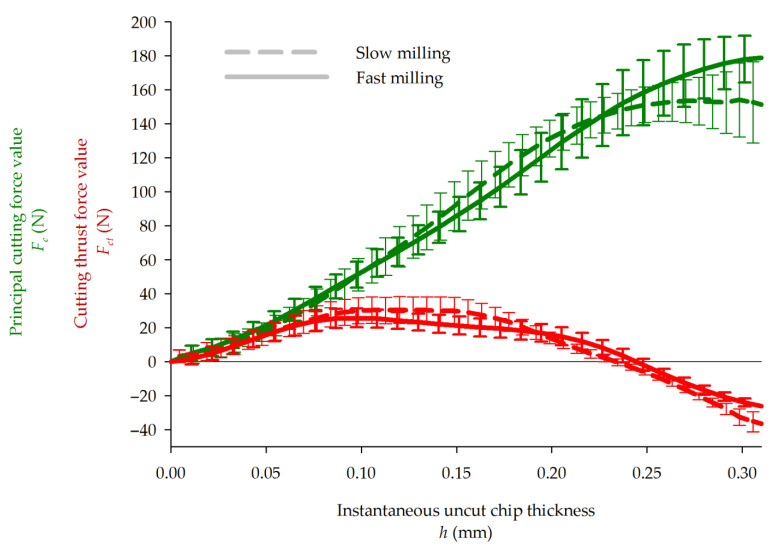
Principal (green) and thrust (red) cutting forces in function of IUCT.

**Figure 11:**
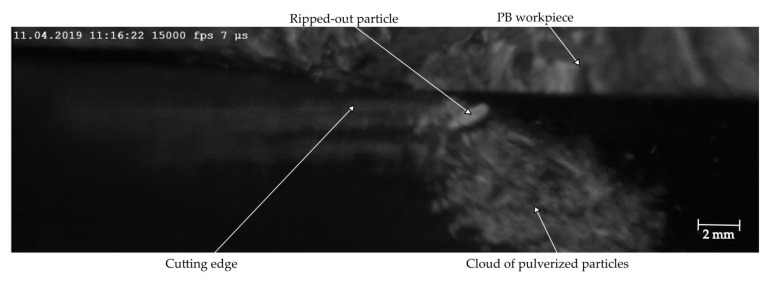
An oversized particle ripped out from the PB structure at the final stage of chip formation is removed within the cloud of small pulverized particles coming from a single uncut chip.

From the plot in Figure 10, it can be observed that the principal cutting force generally grows with the IUCT to achieve the maximum at 179 N in fast and 152 N in slow cutting. The principal cutting force curve flattens at the final stage of single cutting for slow milling. It occurs even when the thinner end-portion of the chip (the decreasing part of IUCT visible in Figure 9) is not taken into consideration. A similar phenomenon was observed earlier, for the maple wood cutting [26]. A hypothesis was formulated that this happens when the tensile strength of the workpiece is exceeded, before the knife reaches the theoretical maximum chip thickness position. In the case of PB up-milling, this idea also seems to be valid. This is shown at the top of Figure 11, where the PB workpiece shows an uncut edge on the right hand, with the cut arc on the left-hand side. The cut chip is not compact but is highly pulverized and is removed from the cutting zone in the form of a cloud. Within the cloud, one single particle, significantly bigger than the others, is visible near the cutting edge. Due to PB’s structure, at the final stage of cutting, single particles are observed to be ripped out from the workpiece, instead of being cut. This happens at the end phase of cutting, because no more material exists to support the chip being cut. This has two consequences: since a portion of the material is ripped out before the end of the theoretical cutting path, the increase in force may be stopped or even reduced. On the other hand, the portion ripped out leaves a void at the end of the next uncut chip, which will surely diminish forces on the next iteration. This phenomenon is less visible when a higher cutting speed is used, which may be caused by a better stress concentration [40]. An increase in standard deviation at the end phase of slow cutting due to the increased frequency of ripping out occurs.

The cutting thrust force curves have both similar courses and are specific for the sharp tool [41]. From the beginning of cutting, up to around 0.075 mm of the uncut chip thickness, both fast and slow cutting processes reveal similar cutting force courses. In this range, the cutting thrust forces increase monotonically, pushing the workpiece away from the tooltip. This is because the rounded tool’s edge rubs the cut surface. On the clearance face of the knife, the chip formation invokes a pulling action on the workpiece. At some point, the pushing action is stabilized, while the pulling forces are increased with the IUCT. For the inspected cases, both actions equilibrate at around 0.24 mm of chip thickness (77% of its maximal value), demonstrating no thrust force. Afterwards, the pulling component of the force overrides pushing, and the workpiece is pulled in the direction of the tool.

### 3.4. Specific Cutting Forces

To compare the cutting forces obtained in this study to other materials and cutting processes, it is convenient to derive specific cutting forces, i.e., related to area of the chip cross–section. A principal specific cutting force *k_c_* and a specific cutting thrust force *k_ct_* are given as:

(11) $$k_{c}=\frac{F_{c}}{bh},\;k_{ct}=\frac{F_{ct}}{bh}$$

**Figure 12:**
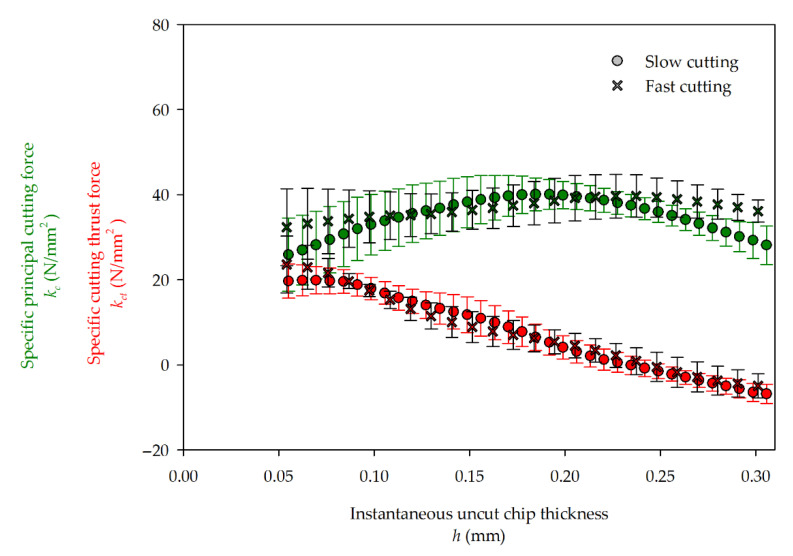
Specific principal (green) and thrust (red) cutting forces in function of IUCT.

These are plotted in Figure 12. Since, at the first stage of cutting, the IUCT is very small and, in reality, the chip does not yet exist (just the rubbing phase), this stage is neither plotted nor taken into account for further consideration. Despite the specific principal cutting force, which was recognized to be nonlinear (at least for wood) [9], from current research, it seems that, to some extent, that the *k_c_* may be considered constant. For both cutting speeds, delicate fluctuations are shown from mean values of 32.0 N/mm^2^ for slow and 37.6 N/mm^2^ for fast cutting. The *k_c_* is less dependent on the IUCT for fast cutting than for the slow one. In general, 60 m/s speed increased the average value of 17.5% compared to 40 m/s. These values are of the same magnitude that was presented for MDF [42]. Only for a medium chip thickness (0.12–0.2 mm) does the specific principal cutting force in slow milling have a slightly higher value than that shown for a higher cutting speed. The specific cutting thrust forces in slow $k_{ct\;1\;}$ and in fast $k_{ct\;2\;}$ milling in the considered range of IUCT decrease linearly (12), without a noticeable difference between each other.

(12) $$k_{ct\;1\;}=-103,3\;h+24,5,\;k_{ct\;2}=-105,6\;h+25,5$$

## 4. Conclusions

A novel, simple method of high-frequency piezoelectric force signal treatment was proposed and successfully used to separate the original force signal from the vibration that was caused by the previous cutting iteration. This allowed for the analysis of single chip cutting forces under industrial CNC milling conditions.

The resultant forces increased in time for single cuts, together with an increase in chip thickness. For two cutting speeds, 40 and 60 m/s, the maximum resultant forces were 153.9 and 182.7 N, respectively. The resultant forces have been decomposed into the principal and thrust (normal) cutting forces and related to the instantaneous chip thickness. This revealed that, during the development of the chip formation process, these forces develop similarly for fast and slow cutting. The principal cutting force grows with increasing chip thickness, while the thrust force first shows the pushing action before representing the pulling action. This was also observed before for wood milling using a sharp knife. 

The average specific principal cutting force for PB peripheral up-milling is equal to 32.0 N/mm^2^ for slow and 37.6 N/mm^2^ for fast milling. The specific cutting thrust force, in turn, decreases linearly, whilst the instantaneous uncut chip thickness increases.

## Data Availability

Data sharing is not applicable to this article.

## Nomenclature

*b*	mm	Workpiece thickness
*R*	mm	Cutting radius
*Z*		Number of blades in the cutter head
*v*	m/s	Cutting speed
*n*	min^−1^	Rotational speed of the tool
*f_z_*	mm	Feed per tooth
*f*	m/min	Feed speed
*H*	mm	Depth of cut
$f_{s}$	Hz	Force signal sampling frequency
*x*, *y*	mm	Coordinates in perpendicular directions as shown in Figure 2, when they occur as indexes, denote properties in these directions
*F*	N	Force signal (directly measured)
$\hat{F}$	N	Discrete Fourier transform of F signal
*F_g_*	N	Signal (imitating the original vibrations) generated in order to clear the F signal
$F_{clear}$	N	Force F signal cleared from vibrations
*F_r_*	N	Resultant force
*α*	°, rad	Angle between the resultant force and x direction (see Figure 5)
$F_{c}$	N	Principal cutting force
$F_{ct}$	N	Cutting thrust force
*t*	ms, s	Time
$y_{c}\left(x\right)$	mm	Function of tooth path in the workpiece
$y_{c-1}\left(x\right)$	mm	Function of precedent tooth path function
β	°, rad	Angle between the cutting force and x direction (see Figure 5)
*h*	mm	Instantaneous uncut chip thickness (IUCT)
*k_c_*	N/mm^2^	Principal specific cutting force
*k_ct_*	N/mm^2^	Specific cutting thrust force

## Abbreviations

CNC	Computer numerical control
*PB*	Particleboard
*MDF*	Medium density fiberboard
*IUCT*	Instantaneous uncut chip thickness
